# Volunteering motivation and sustainable development awareness as predictors of preservice teachers’ personal social responsibility

**DOI:** 10.3389/fpsyg.2026.1817410

**Published:** 2026-06-24

**Authors:** Duygu Gür-Erdoğan, Gülden Kaya-Uyanık, Özlem Canan Güngören

**Affiliations:** Department of Educational Sciences, Faculty of Education, Sakarya University, Sakarya, Türkiye

**Keywords:** personal social responsibility, preservice teachers, sustainable development awareness, teacher education, volunteering motivation

## Abstract

**Introduction:**

Personal social responsibility is an important construct in teacher education, reflecting individuals’ ethical awareness, civic engagement, and commitment to social and sustainable development.

**Methods:**

This study examined whether preservice teachers’ volunteering motivation—conceptualized as volunteering as commitment and volunteering as duty—and sustainable development awareness predict personal social responsibility. Using a cross-sectional correlational research design, data were collected from 960 preservice teachers enrolled in different teacher education programs. Data were gathered using validated self-report scales measuring personal social responsibility, volunteering motivation, and sustainable development awareness. Pearson correlation analysis and hierarchical multiple regression analysis were conducted to examine the relationships among the variables.

**Results:**

The findings revealed that volunteering as commitment and sustainable development awareness positively predicted personal social responsibility, whereas volunteering as duty negatively predicted personal social responsibility. The final regression model explained 30.8% of the variance in personal social responsibility (*R*^2^ = 0.308, *p* < 0.001). Among the predictors, volunteering as commitment emerged as the strongest predictor. The findings suggest that intrinsically oriented volunteering motivations and sustainability awareness are associated with stronger responsibility-oriented tendencies among preservice teachers, whereas externally driven obligation-based volunteering may be associated with lower levels of personal social responsibility.

**Discussion:**

The study contributes to the literature by clarifying the differential roles of volunteering motivations and sustainability awareness in relation to socially responsible orientations in teacher education.

## 1 Introduction

In recent years, the concepts of volunteerism, sustainable development awareness, and personal social responsibility have received increasing attention in the field of teacher education. These concepts reflect not only individual ethical values but also the capacity of individuals to make meaningful contributions to society and the environment. It is emphasized that students with a sense of social responsibility have the potential to create positive change in their communities, and this potential should be developed through universities (Perić, 2011). Higher Education Institutions (HEIs) have the responsibility to shape the actions of the current generation by considering future social, cultural, economic, and environmental impacts and play a unique role in developing a sustainable society (Dzimińska et al., 2020; Finnveden and Schneider, 2023; Ramos et al., 2015). It has been reported that community service-based courses in universities contribute to students’ personal development and social sensitivity, and that students who participate in such courses demonstrate higher levels of individual social responsibility and civic awareness than those who do not (Lucas Mangas et al., 2021; Boiko et al., 2024).

Volunteering is a central tool for the personal development and enhancement of professional skills (soft skills) of university students (Khasanzyanova, 2017; Mazanec, 2022). The motivations underlying volunteering are often multidimensional, encompassing values, social concern, personal development, career, and social understanding dimensions (Clary et al., 1998; Okun and Schultz, 2003). Conceptualized as a multidimensional construct, volunteering is generally examined under two headings: commitment-based (intrinsic motivation and altruistic intentions) and obligation-based (external expectations, i.e., institutional or societal norms) (Clary et al., 1998; Penner, 2002). Commitment-based volunteering stems from personal values, while social norms or institutional pressures drive obligation-based volunteering. Both forms of volunteering have been associated with increased civic engagement and ethical responsibility (Hyde et al., 2016; Handy and Hustinx, 2009).

Sustainable development is recognized as a global vision, and awareness of sustainable development encompasses individuals’ understanding of the interconnected environmental, social, and economic challenges facing today’s world (UNESCO, 2017; Wals, 2015). It encompasses not only knowledge but also a sense of responsibility and a willingness to act for a sustainable future. Therefore, it plays a crucial role in shaping individuals’ sense of duty to both present and future generations (Olsson et al., 2016; Kagawa, 2007).

In line with the United Nations Sustainable Development Goals (SDGs), teachers are tasked with raising awareness of environmental, economic, and social sustainability issues (UNESCO, 2017), and teachers are expected to develop students’ critical thinking skills and encourage them to become active citizens by adopting sustainability-focused pedagogical approaches in their education processes (Sterling, 2001).

In recent years, numerous studies have shown that aligning teachers’ curricula with the SDGs plays a critical role in ensuring students have access to equitable and inclusive learning opportunities (OECD, 2021; UNESCO, 2023). Recent research has demonstrated that teachers play a central role in developing environmental sustainability awareness, promoting lifestyles compatible with nature, and fostering ecological responsibility in students (Chen et al., 2023; Tejedor et al., 2022). Furthermore, teachers’ integration of critical thinking, social justice, and equity perspectives in their pedagogical practices significantly contributes to students’ awareness of social sustainability (Ribeiro et al., 2023; Dzimińska et al., 2020; Bürgener and Barth, 2018).

Another important dimension of SDG-focused education is guiding students to develop a sense of responsibility and citizenship not only for local but also for global problems; this is strongly emphasized in recent studies through the integration of global competencies into curricula (OECD, 2023; Zhao, 2022). Furthermore, teachers’ adoption of interdisciplinary and project-based learning approaches is a decisive factor in students’ development of 21st-century skills such as critical thinking, collaboration, and problem-solving (Buchanan et al., 2019; Tejedor et al., 2022). Finally, recent studies on STEM-based sustainability education reveal that teachers play a significant mediating role in equipping students with the capacity to produce innovative and sustainable solutions (Tasquier et al., 2022; Toma and García-Carmona, 2021). These findings clearly demonstrate that teachers are key actors in implementing the SDGs through education.

In this context, personal social responsibility, volunteering motivation, and sustainable development awareness represent important dimensions of 21st-century education and key competencies for future educators. Examining the relationships among personal social responsibility, volunteering motivation (in both commitment- and duty-based forms), and sustainable development awareness (including environmental, social, and economic dimensions) may provide important insights into the development of socially responsible educators. Building on these considerations, the present study draws on motivational and sustainability-oriented perspectives to examine the predictive relationships among these constructs.

## 2 Theoretical background and hypothesis development

### 2.1 Personal social responsibility

Personal social responsibility encompasses the attitudes and behaviors of individuals that contribute to the well-being of others and society. It reflects internalized ethical values, civic engagement, and a sense of accountability to future generations (Carroll, 1991; Schwartz and Carroll, 2003). Given their central role in shaping future generations, the development of preservice teachers in these areas is critical to the long-term goals of sustainable societies. Personal social responsibility has emerged as a critical construct in contemporary teacher education, reflecting individuals’ ethical, civic, and social engagement in contributing to a more equitable and sustainable society. Because future educators are expected not only to impart knowledge but also to exemplify responsible citizenship, promoting personal social responsibility among preservice teachers has become a key priority (Schreck and Rauffelder, 2021; Lin et al., 2019).

### 2.2 Volunteering as commitment and personal social responsibility

Volunteering is a topic of particular interest today because it not only contributes to the comprehensive education students need to receive but also allows them to acquire skills such as becoming ethical professionals and having a high sense of social commitment, thus enabling them to develop a cohesive identity and the skills necessary for developing interpersonal relationships (Shantz et al., 2014; Smith et al., 2010). This aligns with the goals set by the United Nations in the 2030 Agenda for Sustainable Development. It is worth emphasizing that volunteering increases individuals’ social participation, fosters social awareness, and reinforces a sense of personal responsibility (Wilson, 2000; Penner, 2002).

Volunteering plays a crucial role in developing social awareness, empathy, and personal responsibility, particularly in professions that emphasize social leadership, such as teaching. For preservice teachers, volunteering is not only a complementary experience but also an important component of professional and ethical development. It provides a way for preservice teachers to engage with real-world social issues, develop interpersonal awareness, and develop a sense of obligation to the communities they serve (Choi et al., 2022; Lin et al., 2019; Schreck and Rauffelder, 2021; Chen et al., 2023; Gallant et al., 2017). Research shows that student volunteering can significantly contribute to both personal development and sustainable community development (Perić, 2011; Rampasso et al., 2021). Volunteering also fosters the development of sustainability competencies through experiential learning and reflection, particularly when integrated into curricula through service-learning approaches (Ribeiro et al., 2023; Alshammari et al., 2023). In crises such as natural disasters or humanitarian emergencies, youth volunteering demonstrates a strong connection between moral motivation and personal responsibility (Sengupta et al., 2023). These experiences not only foster individual agency but also deeply embed civic and ethical awareness in the identities of future educators.

Therefore, volunteering as commitment can be considered an important component of personal social responsibility, especially in teacher education programs that aim to produce socially sensitive and ethical professionals.

### 2.3 Volunteering as duty and personal social responsibility

The predictive relationships among volunteering, sustainable development awareness, and personal social responsibility are grounded in both theoretical and empirical evidence. Research increasingly emphasizes that volunteering, whether intrinsically or extrinsically motivated, is associated with individual responsibility and civic engagement (Farooq et al., 2020; Mazanec, 2022). When embedded in educational settings, these experiences contribute not only to personal development but also to broader SDGs (Moon, 2007; Pless, 2012).

Studies show that students who participate in volunteering initiatives, especially those focused on environmental or social justice, are more likely to develop competencies aligned with personal social responsibility, such as ethical reasoning, systems thinking, and social empathy (Puiu and Udriștioiu, 2023; Chen et al., 2023). Furthermore, when such activities are combined with sustainability education, the interaction effect appears stronger than the effect of either factor alone (Rampasso et al., 2021).

### 2.4 Sustainable development awareness and personal social responsibility

To achieve SDGs, universities integrate three core missions: education, research, and community engagement (Compagnucci and Spigarelli, 2020; Dzimińska et al., 2020). Sustainable development education aims to develop individuals’ ability to critically assess current and future environmental and social impacts (Chen et al., 2023). This requires students to acquire sustainability competencies such as systemic thinking, foresight, strategic ability, and comprehensive problem-solving (Finnveden and Schneider, 2023). Instilling sustainable behavior in students is achieved through students’ freedom to act within natural and social conditions. In addition to this freedom, individuals’ knowledge, perceptions, and values are crucial for sustainable development (UNESCO, 2017).

Particularly within the framework of sustainable development education, it is increasingly recognized as a fundamental element of personal social responsibility. According to the sustainable development education framework, education should develop students’ capacities to reflect on their values, understand global challenges, and contribute to achieving sustainability goals (UNESCO, 2017; Tilbury, 2011). Sustainable development awareness has been shown to improve students’ ethical reasoning skills and sense of responsibility towards future generations (Olsson et al., 2016; Dzimińska et al., 2020). Studies in various educational contexts indicate that integrating sustainability into formal curricula supports active citizenship and long-term social engagement (De Andreotti, 2014; Nordén and Avery, 2021). Service-learning models, in particular, are effective in helping students internalize sustainability principles through reflective practice and community engagement (Ribeiro et al., 2023; Alshammari et al., 2023). Research shows that individuals with higher levels of sustainable development awareness are more likely to exhibit prosocial behaviors and develop a stronger sense of personal social responsibility (Olsson et al., 2016; Brundiers et al., 2020). Empirical evidence from China suggests that university-based volunteer activities significantly contribute to attitudes and behavioral intentions related to sustainability, thereby strengthening the synergy between civic engagement and sustainability awareness (Chen et al., 2023). Similarly, in Turkey, initiatives aimed at integrating sustainable development into teacher education have been found to have positive effects on preservice teachers’ values, attitudes, and sense of responsibility (Karaarslan and Teksöz, 2016; Atmaca et al., 2019). Developing sustainable development awareness in teacher education is important not only for personal development but also for preparing educators to integrate sustainability principles into their teaching practices. Preservice teachers with a well-developed awareness of sustainability are more likely to foster critical thinking, participatory learning, and social action among their students (Boeve-de Pauw and Van Petegem, 2010; Evans et al., 2017). These findings suggest that sustainable development awareness is a key determinant of personal social responsibility, particularly when educational experiences foster active, ethical, and interdisciplinary engagement with real-world issues. Therefore, fostering sustainable development awareness in teacher education is not only a pedagogical goal but also a societal imperative. Preservice teachers who acknowledge their roles as both educators and community leaders may view sustainability not only as a professional responsibility but also as a personal moral imperative.

### 2.5 Research hypotheses

Based on the theoretical and empirical literature, volunteering motivation and sustainable development awareness are expected to be associated with preservice teachers’ personal social responsibility. In particular, commitment-based volunteering may strengthen ethical engagement and civic responsibility, whereas duty-based volunteering may be associated with weaker responsibility orientations. Similarly, sustainable development awareness may contribute to greater sensitivity toward social, environmental, and ethical issues. Accordingly, the following hypotheses were developed:

*H1*: Volunteering as commitment positively predicts preservice teachers’ personal social responsibility.

*H2*: Sustainable development awareness positively predicts preservice teachers’ personal social responsibility beyond volunteering as commitment.

*H3*: Volunteering as duty negatively predicts preservice teachers’ personal social responsibility beyond volunteering as commitment and sustainable development awareness.

## 3 Method

This study employed a cross-sectional correlational research design to examine the predictive relationships among preservice teachers’ volunteering motivation, sustainable development awareness, and personal social responsibility. Correlational research designs aim to identify the existence, direction, and magnitude of relationships among variables without manipulating them (Fraenkel and Wallen, 2006; Creswell and Creswell, 2017). In the present study, volunteering as commitment, volunteering as duty, and sustainable development awareness were examined as predictor variables of personal social responsibility.

### 3.1 Participants

The participants of the study consisted of 960 preservice teachers enrolled in different teacher education programs at a faculty of education in Türkiye during the 2025 academic year. Participants were recruited through convenience sampling based on voluntary participation and the data were collected in person from students enrolled in teacher education programs. Data were collected from preservice teachers studying at different grade levels and departments during the data collection period. Prior to data collection, a power analysis was conducted using G*Power 3.1 (Faul et al., 2007) to determine the required sample size. For a hierarchical multiple regression analysis with three predictors, a medium effect size (*f*^2^ = 0.15), a significance level of *α* = 0.05, and a statistical power of 0.95, the minimum required sample size was estimated at 119 participants. The present sample of 960 preservice teachers substantially exceeds this threshold, indicating that the study was adequately powered to detect expected effect sizes. During data screening, questionnaires with missing responses were identified and excluded prior to analysis. All 960 forms retained for analysis were fully completed, with no missing data on any of the study variables. Demographic information for the study group is presented in Table 1.

**Table 1:** Demographic characteristics of the study *group.*

Variables	Category	*N*	%
Gender	Woman	774	80.6
	Male	186	19.4
Department	Science teaching	94	9.8
	Social studies teaching	77	8.0
	Guidance and psychological counseling	204	21.3
	Preschool teaching	111	11.6
	Special education	109	11.4
	English language teaching	125	13.0
	Turkish language teaching	39	4.1
	Math teaching	98	10.2
	Classroom teaching	103	10.7
Class	1st grade	185	19.3
	2nd grade	302	31.5
	3rd grade	218	22.7
	4th grade	255	26.6
Total		960	100

When Table 1 is examined, 80.6% of the participants are female, and 19.4% are male. The participants are studying in nine different departments of the Faculty of Education. Additionally, 19.3% of the participants are in their first year, 31.5% are in their second year, 22.7% are in their third year, and 26.6% are in their fourth year.

### 3.2 Data collection tool

Data were collected using four self-report measurement instruments assessing sustainable development awareness, personal social responsibility, volunteering as commitment, and volunteering as duty. Higher scores on the scales indicated higher levels of the related construct.

#### 3.2.1 Sustainable development awareness scale

The scale developed by Atmaca et al. (2019) consists of three sub-dimensions, namely economy, society, and environment, and a total of 36 items. Items are rated on a five-point Likert-type scale ranging from 1 (Strongly Disagree) to 5 (Strongly Agree), with total scores ranging from 36 to 180; higher scores reflect greater sustainable development awareness. A sample item is “I pay attention to the sustainability of natural resources in my daily life.” The validity of the scale was examined using confirmatory factor analysis. The fit indices were found as *χ*^2^ = 964.497, df = 575, *p* = 0.000, χ^2^/df = 1.677, RMSEA = 0.40, S-RMR = 0.44, AGFI = 0.871, GFI = 0.889, IFI = 0.931, TLI = 0.923. The Cronbach’s alpha reliability coefficient for the overall scale was calculated as 0.91. The reliability value of the scale for this study was 0.898.

#### 3.2.2 Personal social responsibility scale

The scale, developed by Davis et al. (2020) and adapted to our culture by Koçar and Seki (2023), consists of five subdimensions and a total of 19 items. Items are rated on a ten-point Likert-type scale ranging from 1 (Strongly Disagree) to 10 (Strongly Agree), with total scores ranging from 19 to 190; higher scores indicate higher levels of personal social responsibility. A sample item is “I feel personally responsible for helping others in need.” The validity analyzes of the scale were examined with confirmatory factor analysis, and the fit indices were found to be χ^2^/df = 1.91, RMSEA = 0.06, SRMR = 0.06, AGFI = 0.85, GFI = 0.90, NFI = 0.90, IFI = 0.93, CFI = 0.94, and TLI = 0.93. The Cronbach’s alpha reliability coefficient for the overall scale was calculated as 0.88. The reliability value of the scale for this study was 0.838.

#### 3.2.3 Volunteering as commitment scale

The scale, developed by Gallant et al. (2017) and adapted to our culture by Gürbüz et al. (2022), consists of a single dimension and nine items. Items are rated on a seven-point Likert-type scale ranging from 1 (Strongly Disagree) to 7 (Strongly Agree), with total scores ranging from 9 to 63; higher scores reflect a stronger commitment-based volunteering orientation. A sample item is “Volunteering is an important part of who I am.” The validity analyses of the scale were examined with confirmatory factor analysis, and the fit indices were found to be χ^2^/df = 3.3, RMSEA = 0.09, AGFI = 0.88, GFI = 0.95, NFI = 0.96, CFI = 0.97, and TLI = 0.93. The Cronbach’s alpha reliability coefficient for the overall scale was calculated as 0.89. The reliability value of the scale for this study was 0.951.

#### 3.2.4 Volunteering as duty scale

The scale, developed by Gallant et al. (2017) and adapted to our culture by Gürbüz et al. (2022), consists of a single dimension and nine items. Items are rated on a seven-point Likert-type scale ranging from 1 (Strongly Disagree) to 7 (Strongly Agree), with total scores ranging from 9 to 63; higher scores reflect a stronger duty-based volunteering orientation. A sample item is “I feel obligated to volunteer because it is expected of me.” The validity analyses of the scale were examined with confirmatory factor analysis, and the fit indices were found to be χ^2^/df = 2.84, RMSEA = 0.08, AGFI = 0.88, GFI = 0.95, NFI = 0.97, CFI = 0.98, and TLI = 0.96. The Cronbach’s alpha reliability coefficient for the overall scale was calculated as 0.93. The reliability value of the scale for this study was 0.918.

### 3.3 Procedure

Prior to data collection, ethical approval for the study was obtained from the Educational Research and Publication Ethics Committee of Sakarya University. After obtaining institutional permission, the data collection process was carried out with preservice teachers enrolled in different teacher education programs.

Participants were informed about the purpose of the study, and participation was based on voluntary consent. Data were collected through self-report questionnaires administered during the data collection period. Participants were assured that their responses would remain anonymous and confidential, and no personally identifiable information was collected. Only fully completed forms were included in the analyses.

### 3.4 Data analysis

The aim was to reveal the effect levels based on the relationship between preservice teachers’ personal social responsibilities (PSR), volunteering as commitment(OVC), and volunteering as duty(OVD), and their sustainable development awareness(SDA). For this purpose, the relationship between the variables was revealed using the Pearson moment correlation coefficient, and the effect levels were revealed using multiple regression analysis. Prior to regression analysis, several assumptions were examined. The normality of the distribution of the variables was examined using the Kolmogorov–Smirnov normality test, and it was found that the distributions for all variables bore the normality property (KSPSR = 9.037, *p* > 0.05; KSSDA = 8.087, *p* > 0.05; KSOVC = 7.075, *p* > 0.05; KSOVD = 10.089, *p* > 0.05). Homoscedasticity was assessed through visual inspection of residual scatterplots, which revealed no systematic patterns indicative of heteroscedasticity. Residual diagnostics indicated that the assumption of independence of errors was satisfied (Durbin-Watson = 1.94). Influential cases were examined using Cook’s distance values, all of which fell well below the conventional threshold of 1.0 (maximum Cook’s D < 0.05), indicating that no individual observation exerted undue influence on the regression solution. Leverage values were also within acceptable limits. To address potential common-method bias arising from the single-source, self-report design, Harman’s single-factor test was conducted. The unrotated factor solution yielded a first factor accounting for less than 50% of the total variance, suggesting that common-method bias is unlikely to constitute a serious threat to the validity of the findings. The SPSS 27 package program was used for all analyses.

## 4 Findings

The personal social responsibility scores of the 960 preservice teachers who participated in the study, their volunteering as commitment, volunteering as duty, and their sustainable development awareness were examined using simple correlation analysis. Table 2 presents descriptive statistics and correlation values for the variables examined in this study.

**Table 2:** Arithmetic mean, standard deviation, and correlation values showing relationships between variables for scores obtained from personal social responsibility (PSR), volunteer as commitment (OVC), volunteer as duty (OVD), and sustainable development awareness (SDA) scales.

Variables	$\bar{X}$	SS	PSR	1	2	3
PSR	134.4208	24.20688				
Predictor variables						
1. SDA	157.8500	14.15829	0.358**			
2. OVC	46.9302	11.18814	0.502**	0.280**		
3. OVD	25.0948	11.92435	−0.338**	−0.287**	−0.476**	

**p < 0.001.

As shown in Table 2, personal social responsibility was positively and moderately correlated with sustainable development awareness (*r* = 0.358, *p* < 0.01) and volunteering as commitment (*r* = 0.502, *p* < 0.01). In contrast, personal social responsibility was negatively and moderately correlated with volunteering as duty (*r* = −0.338, *p* < 0.01).

Personal social responsibility was predicted by the variables of volunteering as commitment, volunteering as duty, and sustainable development awareness. In multiple regression analysis, statistically significant correlations between the independent variables and the dependent variable, and relationships between the independent variables not exceeding 0.80, provide evidence that regression analysis can be conducted on these variables (Büyüköztürk, 2006). The findings in Table 2 for this study indicate that multiple regression analysis can be conducted. Furthermore, to check the assumption of multicollinearity, one of the fundamental assumptions of multiple regression analysis, the Variance Increment Factor (VIF), Tolerance Value, and Condition Index (CI) values were examined. The results are presented in Table 3.

**Table 3:** Coefficient table for multicollinearity assumption.

Variables	VIF	Tolerance value	CI
Constant			1.000
SDA	1.122	0.891	4.487
OVC	1.331	0.751	12.992
OVD	1.337	0.748	23.358

For the multicollinearity assumption, the VIF value is expected to be less than 10, the Tolerance value to be greater than 0.10, and the CI to be less than 30 (Hair et al., 2006; Tabachnick et al., 2001; Uyanık and Güler, 2013). When the values in Table 3 are examined in light of all these limits, it is evident that there is no multicollinearity problem in the data set used in the study and that the data are suitable for multiple linear regression analysis.

To determine the predictive power of preservice teachers’ volunteering as commitment, volunteering as duty, and sustainable development awareness on their personal social responsibility scores, the predictive contributions of predictor variables on personal social responsibility was examined using the hierarchical regression technique of multiple regression analysis. In the hierarchical regression analysis, predictor variables were entered into the model in successive steps based on theoretical considerations regarding their relationships with personal social responsibility. Volunteering as commitment was entered in the first step, sustainable development awareness was added in the second step, and volunteering as duty was included in the final step to examine their additional predictive contributions to personal social responsibility. The results are presented in Table 4.

**Table 4:** Prediction level of personal social responsibility (PSR) by volunteering as commitment (OVC), volunteering as duty (OVD), and sustainable development awareness (SDA).

Model	Predictor variables	B	Standard error	*β*	*t*
1	Constant	83.484	2.917		28.616**
	OVC	1.085	0.060	0.502	17.948**
		*R* = 0.502	*R*^2^ = 0.252 Adj. *R*^2^ = 0.251, Δ*R*^2^ = 0.252	*F*_(1, 958)_ = 322.146, Δ*F* = 322.146	*p* < 0.001
2	Constant	26.513	7.349		3.608**
	OVC	0.943	0.061	0.436	15.496**
	SDA	0.403	0.048	0.236	8.393**
		R = 0.550	R^2^ = 0.303, Adj. R^2^ = 0.301, ΔR^2^ = 0.051	*F*_(2, 957)_ = 207.969, ΔF = 70.442	*p* < 0.001
3	Constant	38.214	8.517		4.487**
	OVC	0.865	0.067	0.400	12.879**
	SDA	0.379	0.049	0.222	7.786**
	OVD	−0.170	0.063	−0.084	−2.693**
		*R* = 0.555	*R*^2^ = 0.308, Adj. *R*^2^ = 0.306, Δ*R*^2^ = 0.005	*F*_(3, 956)_ = 141.970, Δ*F* = 7.253	*p* < 0.001

Dependent variable: personal social responsibility-PSR, **p < 0.001.

Upon examining the findings in Table 4, it is evident that the regression model (Model 3), which includes the variables of volunteering as commitment, sustainable development awareness, and volunteering as duty, is statistically significant [*F*_(3, 956)_ = 141.970, *p* < 0.001]. According to the results of the hierarchical regression analysis, three steps were included in the multiple regression analysis. The predictor variable of volunteering as commitment, which was processed in the first step of the regression analysis, explains 25.2% of the total variance regarding personal social responsibility *R* = 0.502, *R*^2^ = 0.252, Adj. *R*^2^ = 0.251, Δ*R*^2^ = 0.252, Δ*F* = 322.146, *p* < 0.001). In the second step of the hierarchical regression analysis, the variable of sustainable development awareness was added to the model, along with the variable of volunteering as commitment. The variables of volunteering as commitment and sustainable development awareness, together, can explain 30.3% of personal social responsibility (*R* = 0.550, *R*^2^ = 0.303, Adj. *R*^2^ = 0.301). In this case, it can be said that the variable of sustainable development awareness contributes 5.1% to the equation (Δ*R*^2^ = 0.051, Δ*F* = 70.442, *p* < 0.001). With other variables held constant, the Beta coefficient for volunteering as a commitment variable was calculated to be 0.436, and the Beta coefficient for the sustainable development awareness variable was calculated to be 0.236. The *t*-values for both variables were determined to be statistically significant (*t* = 15.496, *t* = 8.393, *p* < 0.001, respectively). In the third step of the hierarchical regression analysis, volunteering as duty variable was included in the model along with volunteering as commitment and sustainable development awareness variables. Volunteering as commitment, sustainable development awareness, and volunteering as duty variables together explain 30.8% of the total variance related to personal social responsibility (*R* = 0.555, *R*^2^ = 0.308, Adj. *R*^2^ = 0.306). Accordingly, it can be said that the volunteering as duty variable contributes 1% to the regression Accordingly, it can be said that volunteering as duty variable contributes 1% (Δ*R*^2^ = 0.005) to the regression equation. In this step, the beta coefficients for the variables volunteering as commitment, sustainable development awareness, and volunteering as duty were calculated as 0.400, 0.222, and −0.084, respectively. The *t*-values for volunteering as commitment and sustainable development awareness were statistically significant at *p* < 0.001 (*t* = 12.879 and *t* = 7.786, respectively), and volunteering as duty was also statistically significant (*t* = −2.693, *p* = 0.007).

*R*^2^ values for the variables volunteering as commitment, sustainable development awareness, and volunteering as duty were examined, it was determined that volunteering as commitment was the first variable to predict the personal social responsibility status of preservice teachers, sustainable development awareness was the second variable, and volunteering as duty was the third variable to predict the personal social responsibility status of preservice teachers at a negative level. According to the results of the multiple regression analysis, the regression equation for predicting the personal social responsibility status of preservice teachers is as follows:

$$\begin{array}{l} \text{Personal Social Responsibility}=38.214+0.865\\ {}*(\text{Volunteering}\;\mathrm{as}\;\text{Commitment})+0.3794\\ {}*(\text{Sustainable Development Awareness})+(-0.17)\\ {}*(\text{Volunteering}\;\mathrm{as}\;\text{Duty}). \end{array}$$

## 5 Conclusion and discussion

This study examined the predictive relationships among preservice teachers’ volunteering as commitment, volunteering as duty, sustainable development awareness, and personal social responsibility. As a result of the correlation and multiple linear regression analyses, it was determined that there were a statistically significant relationship between personal social responsibility and volunteering as commitment, volunteering as duty, and sustainable development awareness. The personal social responsibility status of preservice teachers was statistically significantly predicted by the variables of volunteering as commitment at a positive level in the first place, sustainable development awareness at a positive level in the second place, and volunteering as duty at a negative level in the third place.

An examination of studies on personal social responsibility, the dependent variable of the study, reveals that universities play a critical role in developing the personal social responsibility levels of students, including preservice teachers (Żak et al., 2023; Boiko et al., 2024). Personal social responsibility is the primary responsibility an individual assumes towards their family, workplace, community, and environment (Żak et al., 2023). In this context, volunteering and awareness of the SDGs stand out as key variables that may contribute to explaining and strengthen preservice teachers’ personal social responsibility.

The finding that volunteering as commitment positively predicted personal social responsibility indicates that intrinsic commitment to volunteer activities may strengthen an individual’s perception of social responsibility. This result is consistent with the literature, which suggests that volunteering increases prosocial behaviors and social contribution orientations (Smith et al., 2010). Because volunteering as commitment is associated with the sense of connectedness and positive outcomes that an individual derives from the volunteering experience (Gallant et al., 2017), it appears that volunteers are generally driven by internal drivers, such as personal satisfaction, development, and emotional attachment to the organization. The strongest driver for volunteering is value motivation, which describes individuals’ desire to express values such as altruism or humanitarian concerns, which are associated with volunteering as commitment (Shantz et al., 2014; Breitsohl and Ehrig, 2017). The relationship between values, motivation, and volunteer time spent is fully mediated by volunteer engagement. Participation allows volunteers to express their prosocial values, leading them to dedicate more time to their cause. In other words, volunteers with strong value motivation are more likely to participate and dedicate more time to volunteering because it allows them to feel authentic and express their preferred self.

Furthermore, the relationship between volunteer participation and time spent is strengthened by volunteers’ commitment to beneficiaries (Shantz et al., 2014). Volunteers’ values that align with organizational values increase job satisfaction and the time spent volunteering (Hoye and Kappelides, 2021). Participation allows them to channel physical, cognitive, and emotional energy into volunteering activities (Shantz et al., 2014).

Volunteering is a fundamental way for preservice teachers to embody their social responsibilities, and participation in volunteering activities fosters a sense of agency and responsibility in preservice teachers (Boiko et al., 2024). Particularly at the university level, the implementation of structured forms of volunteering, such as Community Service Practices courses and Service-Learning projects, clearly demonstrates a trend toward improving students’ norms of personal responsibility for sustainable development (the ethical and moral responsibility components of personal social responsibility) (Lucas Mangas et al., 2021; Villacé-Molinero et al., 2025). Furthermore, students may be motivated to volunteer for reasons such as building a career and helping others (Boiko et al., 2024; Smith et al., 2010). These activities also develop the necessary life skills and professional experience in students (Alshammari et al., 2023; Boiko et al., 2024), while volunteers gain methodological competencies such as project planning and implementation (Brundiers et al., 2020; Villacé-Molinero et al., 2025), which may further strengthen the sense of responsibility that is vital for future educators.

Another important finding of the study is that sustainable development awareness significantly predicted personal social responsibility. This suggests that individuals with sustainability awareness may be more sensitive to social, environmental, and economic issues, and that this sensitivity may strengthen their understanding of social responsibility (Biasutti and Frate, 2017; Wu and Shen, 2016). Increasing SDG awareness, a cognitive predictor that determines the direction of personal social responsibilities towards global and sustainable goals, may contribute to improving individuals’ pro-sustainable behaviors (Yamane and Kaneko, 2021). Ensuring the formation of values and attitudes compatible with sustainable lifestyles through the development of sustainability-related knowledge and competencies is considered a central responsibility of higher education institutions (Brundiers et al., 2020; UNESCO, 2017; Rodríguez-Zurita et al., 2025). Higher education students’ awareness of the SDGs and their acquisition of knowledge about these goals raise their sustainability awareness at the individual level (Yamane and Kaneko, 2021). Awareness of the SDGs is often conceptualized as a prerequisite or the first stage of learning outcomes (LOs), which are closely related to personal social responsibility and include students’ values, attitudes, and agency (Probst, 2022). Cognitive-behavioral incentives and awareness training strengthen the values that underlie personal social responsibility towards meeting social, ecological, and economic interests in all members of the university community (and therefore in preservice teachers) (Żak et al., 2023). Increased awareness of the SDGs directly affects the key components that constitute students’ personal social responsibility: attitudes, values, and commitment (Svanström et al., 2008; Shephard, 2008; Rodríguez-Zurita et al., 2025). The most tangible outcome of SDG awareness in terms of personal social responsibility is the development of students’ capacity to engage in actions that lead to social change (Probst, 2022). The most effective way to translate SDG awareness into action is through experiential learning approaches. In this context, service-learning and community engagement are valuable approaches for students and teachers to increase their awareness, understanding, and participation in the SDGs (Rodríguez-Zurita et al., 2025). Increasing preservice teachers’ knowledge and awareness of sustainable development may help them become more responsible individuals both in their professional lives and in their roles in society. This result also supports UNESCO (2020) reports, which indicate that sustainable development education should play a central role in teacher training programs.

One of the striking findings of the study is that volunteering as duty negatively predicted personal social responsibility. This finding suggests that individuals who view volunteering as an obligation, liability, or responsibility may have lower perceptions of social responsibility. The literature indicates that volunteering driven by intrinsic motivation is more strongly associated with social responsibility and prosocial behaviors, whereas volunteering activities that constitute the dimensions of volunteering as duty may be undermined by structural or interpersonal constraints imposed by volunteering activities that prevent participation in leisure time or other activities (Gallant et al., 2017); normative commitment, which describes a sense of loyalty based on a perceived obligation to an organization (Valéau et al., 2013); civic and moral obligations (Matsuba et al., 2007); cultural obligations (Tamasese et al., 2010); and external obligations (Clary et al., 1998; Bang and Ross, 2009). These findings may suggest that obligation-based volunteering motivations are associated more with external expectations and compliance than with internalized responsibility orientations. From this perspective, perceiving volunteering as “duty” may negatively affect the individual’s level of personal social responsibility by reducing their self-directed social contribution motivation.

Overall, the findings reveal that preservice teachers’ personal social responsibility levels are associated with both their tendencies to volunteer and their sustainable development awareness. While awareness of the SDGs serves as a prerequisite for positively influencing students’ attitudes, values, and social commitments toward sustainability issues (the core components of personal social responsibility), this awareness alone is not sufficient. The shallowness of SDG awareness (the “shallow consensus” approach, which dominates the literature on sustainable development education in higher education) may prevent students from translating this knowledge into disruptive and transformative social action, a critical dimension of personal social responsibility (Probst, 2022). Therefore, the transformation of personal social responsibility into a sustainable outcome is closely linked to the dimensions of obligation that become evident in volunteering (Shantz et al., 2014). To achieve an enhanced level of personal social responsibility in line with the global change envisioned by the SDGs, universities should not only raise awareness but also foster experiential learning approaches and value alignment, enabling students to develop a social commitment based on emotional attachment to the organization’s cause that transcends duty-based obligations (Hoye and Kappelides, 2021; Rodríguez-Zurita et al., 2025). Consequently, the practical experiences provided by universities through volunteering activities and the conscious training provided on the SDGs create a strong tendency for preservice teachers to develop personal social responsibility norms; this, in turn, enables them to become active change agents who disseminate the SDGs to society in their future roles (UNESCO, 2017; Brundiers et al., 2020). This result highlights the importance of incorporating activities that strengthen volunteering through intrinsic motivation, sustainable development education, and community service practices into teacher training programs. In particular, developing volunteering as a commitment-based value rather than a sense of obligation can contribute more strongly to students’ sense of social responsibility. Furthermore, the findings support recommendations for increasing sustainability-based content in education faculties by revealing that sustainable development awareness is a critical component in preservice teachers’ understanding of social responsibility.

In light of the findings, recommendations for practice and research can be offered to deepen preservice teachers’ knowledge of their personal social responsibility. First, to transform awareness of the SDGs from abstract knowledge to concrete, action-based personal social responsibility, the integration of experiential pedagogies, such as Service-Learning and Community Engagement, into university curricula should be encouraged. These approaches may increase students’ awareness, understanding, and participation in the SDGs, fostering their social commitment while also enhancing sustainability competencies, such as systems thinking and critical thinking. For future research, given that existing studies primarily focus on knowledge and awareness, it is crucial to operationalize and measure the concept of personal social responsibility in a more integrated manner, encompassing the social, economic, and environmental dimensions of the SDGs. Furthermore, longitudinal research designs should be used to track the transformation of this awareness into behavioral outcomes and competencies. From a pedagogical perspective, detailed studies are needed to compare the relative effectiveness of different learning approaches (service-learning, project-based, etc.) on personal social responsibility and SDG competencies, particularly their impact on affective dimensions (attitudes and values). Finally, based on the finding that the OVD dimension negatively predicts personal social responsibility, strategies should be developed to mitigate the potential negative effects of OVD’s sub-dimensions, such as burden and constraint. Additionally, the influence of these dimensions on students’ initiation and maintenance of SDG-focused personal social responsibility practices should be examined in greater detail.

## 6 Limitations and suggestions

Despite its contributions, this study has several limitations that should be acknowledged. First, the use of a cross-sectional correlational design limits causal interpretations of the relationships among volunteering motivation, sustainable development awareness, and personal social responsibility. Therefore, causal conclusions cannot be drawn from the findings. Longitudinal or experimental designs may provide a more comprehensive understanding of how awareness and motivational orientations are associated with behavioral outcomes over time. Second, reliance on self-report instruments, which may have increased the risk of social desirability bias and common-method bias. Because all variables were collected from the same participants at a single point in time, the findings should be interpreted with caution. Future research may benefit from using multi-method approaches, including observational data, qualitative evidence, or behavioral indicators, to provide a more comprehensive assessment of personal social responsibility.

Furthermore, although the study examined key psychological predictors, the construct could be operationalized more comprehensively by incorporating the social, economic, and environmental dimensions of the Sustainable Development Goals. Comparative research is also needed to evaluate the relative effectiveness of different pedagogical approaches, such as service-learning and project-based learning, may also contribute to the literature on teacher education and sustainability competencies. Finally, given the negative predictive role of duty-based volunteering motivation, future studies should explore the underlying mechanisms—such as perceived burden or constraint. In addition, cross-cultural and longitudinal studies may provide deeper insight into how volunteering motivations and sustainability awareness are associated with socially responsible orientations across different educational contexts.

## Data Availability

The original contributions presented in the study are included in the article/supplementary material, further inquiries can be directed to the corresponding author.
